# Variations in intraocular pressure and visual parameters before and after using mobile virtual reality glasses and their effects on the eyes

**DOI:** 10.1038/s41598-022-07090-x

**Published:** 2022-02-24

**Authors:** Ching-Huang Lin, Hsien-Chang Lin, Chien-Yu Chen, Chong-Chung Lih

**Affiliations:** 1 Department of Electronic Engineering, National Yunlin University of Science and Technology, Yunlin, Taiwan 640; 2 Graduate School of Engineering Science and Technology, National Yunlin University of Science and Technology, Yunlin, Taiwan 640; 3 Graduate Institute of Color and Illumination Technology, National Taiwan University of Science and Technology, Taipei, Taiwan 106; 4 Department of Optometry, Jenteh Junior College of Medicine, Nursing and Management, Miaoli, Taiwan 35664

**Keywords:** Eye manifestations, Outcomes research

## Abstract

We examined the effects of using mobile devices with immersive virtual reality for a short period on the physiological parameters of both eyes. The average age of the 50 participants (23 men and 27 women) was 17.72 ± 1.48 years, and refractive error ranged from 0 D to − 5.00 D. All the participants wore + 3.00 D glasses and underwent a 5-min relaxation adjustment through the atomization method. The participants wore immersive virtual reality (VR) glasses to watch a movie on a roller coaster for 10 min. Their relevant physiological parameters of the eyes were measured both before and after using VR glasses. Compared with before VR use, no significant difference (*P* > 0.05) was observed in the near-horizontal vergence and refractive error but a significant difference (*P* < 0.05) was observed in the amplitude of accommodation, intraocular pressure, divergence/convergence, and stereopsis after VR use. The corneal elastic coefficient was > 0.2 MPa, and we used Friedenwald’s eye rigidity relationship to obtain the K value (0.065–0.09). Approximately 10% of the participants experienced cybersickness symptoms such as nausea and dizziness. The use of VR to watch three-dimensional movies reduced intraocular pressure, which may help prevent or treat glaucoma. Moreover, the binocular convergence was higher when viewing near-field objects in VR than in the real world. Therefore, individuals with convergence excess may experience symptoms. Binocular parallax is the most likely cause of cybersickness symptoms. Thus, mobile VR devices with higher quality and comfort are necessary.

## Introduction

Since the launch of Google Cardboard in 2014, users have been inexpensively and conveniently able to experience virtual reality(VR) effects through electronic device, and the product has accelerated the popularity and development of VR. While using mobile devices with immersive VR, the screen is only 15 cm away from the eyes to VR wearing plane, thus leading to the shrinkage of the pupils, contraction of the ciliary muscles, adjustment of the lens, and contract or loosing of extraocular muscles owing to the proximity between the eyes and the screen^1^. The contract or loosing of extraocular muscles affects the comfort of the eyes^2^. In smartphone VR videos, because only binocular vision is working during watching, a certain degree of convergence is necessary to reach fusion, affecting the eyes’ horizontal vergence and stereoscopic ability^3–7^. In addition, users are required to repeatedly change the focus due to constant moving scenes in a VR videos, which might easily lead to changes in intraocular pressure (IOP)^8,9^. Though this type of intraocular pressure change is not long lasting. Therefore, the user does not have to undergo strenuous exercise or changes in blood pressure throughout the body, only the visual focus and visual synthesis of the human brain suffice for coping with IOP changes. The equilibrium of IOP is mainly maintained by changes in the resistance of aqueous humor outflow. The regulation of the secretion and outflow of the aqueous humor is key for maintaining IOP, which is subject to diurnal variation in healthy eyes^10^. To control IOP, the constriction and dilation of pupils are required during aqueous humor circulation which led to the contraction of the ciliary muscles and the expansion of the lens change the refractive and accommodative powers of the eye. However, IOP depends on many other factors that may have a brief transient effect to a much longer-term effect. Factors exerting short-term effects include food or fluid intake, changes in systemic blood pressure, and vigorous physical activity. Although most factors affecting IOP are random and inherently unstable^11^, the resulting short-term effects are observable for a limited period.

In previous studies, the effects of immersive VR devices on visual parameters are all focused on the visual effects after 30 min of use^3–7^. However, it takes about 10 min to get into immersion using VR mobile devices. From the beginning of using VR devices to entering immersion, people are in the transition period of adapting to VR devices. In the Ref.^12,13^ have pount out, the continuous conflict between accommodation and vergence, which stimulates the continuous adaptation of the visual system, indeed cause difficulties in this period. The conflict between accommodation and vergence may cause binocular vision defects. Therefore, examining changes occurring in IOP and related ocular physiological parameters, before and after the use of mobile devices with immersive VR during this period of time can indicate the immediate physiological effects of these products on the eyes. And then help us to design better virtual reality equipment.

In ours study, since the experiment is only 10 min, we expect that IOP will not change too much during this time, which allows us to use a method similar to a small signal analysis^14^ to help analyze the dynamics of short-term IOP change and calculate the elastic modulus of the cornea. In this method, the small variation in IOP has a linear relationship with the eyeball volume. This is mainly because the elastic modulus of the cornea and sclera provides the integrity of the optical function of the eye. In the previous studies have shown^15–17^ that when the lens changes shape with accommodation, the radius of curvature of the cornea as well as the size and shape of the eye ball will change accordingly. However, physiologically relevant results must come from measurements of intact eyes in vivo. This will require non-invasive measurements in the living eye and the detection systems that can distinguish very small changes of strain. Such experiments are not easy to design, measurements are affected by small changes in the dynamics of any life system. In some related alternative experimental methods^18–20^, the variation of IOP is about 5-10 mmHg, at this time, the IOP increase largely, the contribution of extraocular muscles’ pulling effect could not be ignored.

The applicability of mobile devices with immersive VR (i.e., head-mounted display) in a clinical setting remains concerning because it can cause cybersickness symptoms such as nausea, dizziness, disorientation (motion sickness), fatigue, and instability (that is, symptoms and effects caused by VR)^21–26^. In this study, unwanted sense of immersion and spatial dislocation is considered, so there is no need to pay attention to cognitive neuroscience and neuropsychology issues^27–30^. Changes in the basic condition of the eyes was examined considering all problems begin with the eyes. Relevant physiological parameters such as the refractive error, amplitude of accommodation (AA), IOP, near-horizontal vergence (NHV), and stereopsis were also measured, before and after using VR glasses for a short period. Short-term use of VR glasses can help analyze dynamic changes in IOP and use to calculate the corneal elastic modulus.

## Experimental design

### Participants

We recruited 50 participants (23 men and 27 women). The average age of the participants was 17.72 ± 1.48 years^31^, and their refractive error was between 0 and − 5.00 D. All participants were instructed to have an adequate sleep prior to the test, not consume food or beverages containing caffeine or alcohol for 8 h prior to the test, and understand the experimental process of the study. The aim of the study was explained to all the participants, and their consent was obtained at the time of their first clinical visit. This study was approved by the Human Research Ethics Committee of National Cheng Kung University (approval No. NCKU HREC-E-107-315-2) and was conducted in accordance with the Declaration of Helsinki.

### Experimental method

**Figure 1:**
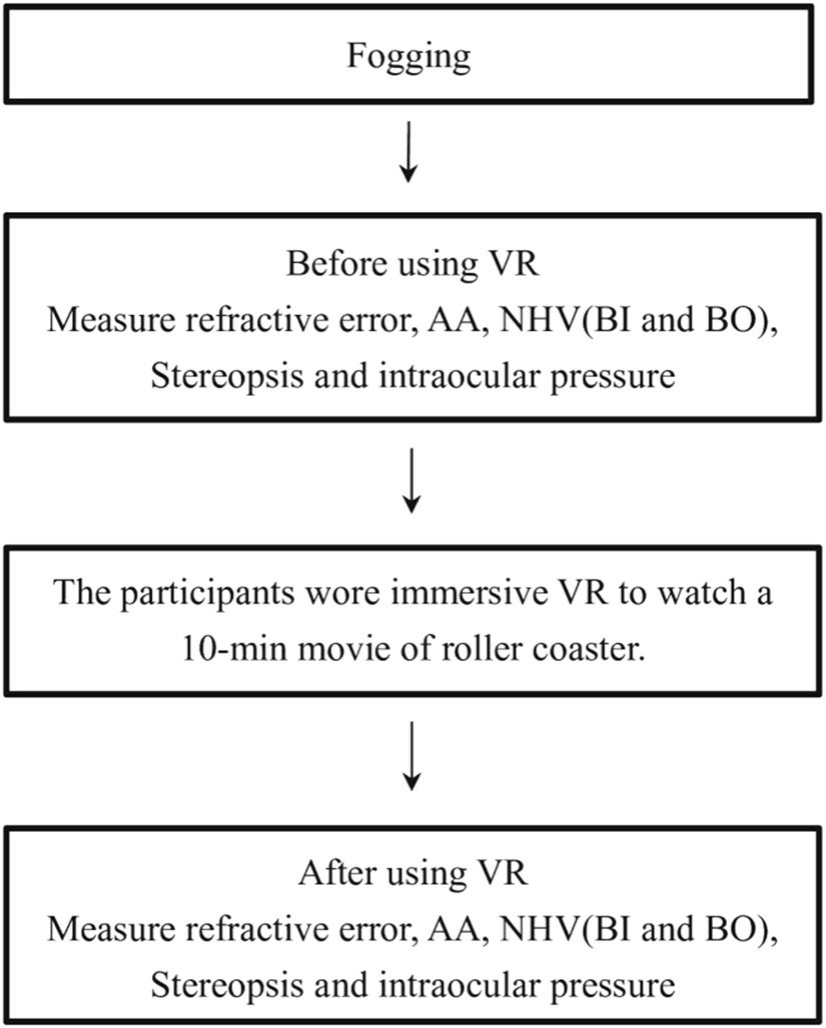
Experimental process.

**Figure 2:**
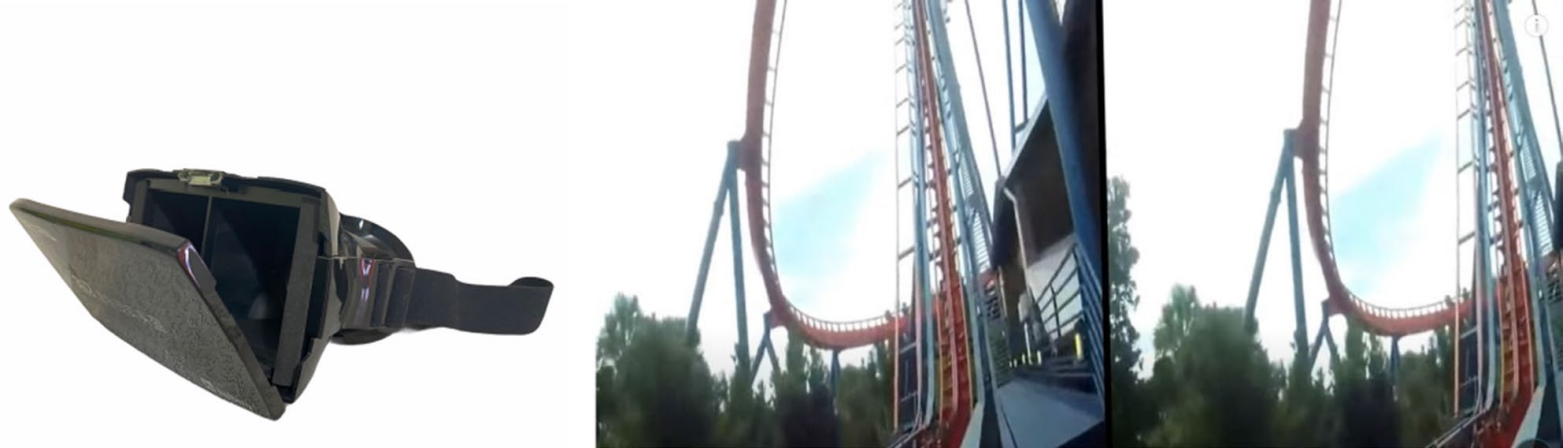
Use of wearable VR glasses.

We want to observe the initial reaction of the eyes when using a VR headset. Therefore, we allowed the participants to use VR only for approximately 10 min^12^ because we did not intend the participants to have a considerable degree of immersion (although the purpose of using VR is to have a sense of immersion). In this case, both eyes respond in the same way for fusion, that is, accommodation and vergence. Regarding the difference in the impact of the film type on the eyes, it should be occurs after the participant enters the immersive state^12^. Personal factors, such as device type, head shape, wearing gestures, etc., will affect the length of time the participant enters the immersive state. Figure 1 depicts the entire experimental process. In the beginning, each participant wore + 3.00 D glasses and used the atomization method for 5 min of relaxation and adjustment before starting the experiment. The physiological parameters of the eyes were evaluated for the first time. Next, the participants were asked to use mobile devices with immersive VR to watch a 10-min movie on a roller coaster, as shown in Fig. 2. The relevant physiological parameters of the eyes were evaluated for the second time for all the participants. The physiological parameters examined are described below.

### Experimental measurement items and equipment

#### Fogging

To generate fog vision, some convex lenses were added in front of the examinee’s eyes, thereby causing artificial nearsightedness; the lenses were added to enable the examinee’s eyes to automatically relax and adjust to the scene. Optometry was subsequently performed to obtain a more objective result. Wearing a convex lens to view a distant scenery reduces the clarity of the scene. When used to generate fog vision, this method is called the cloud method. We used the BICOH JAPAN ADJUSTABLE TRIAL FRAME FOR 547 PD54-70 and + 3.00 D lens.

#### Refractive error examination

We used the SHIN-NIPPON K5001 autorefractor to perform optometry.

#### Accommodation

The lens in the human eye is equivalent to the focusing lens used in the camera. The lens can project the image on the retina. The thickening of the lens caused by the contraction of the ciliary muscle can help judge the object's distance.

In this study, we used the negative lens method in which a near-vision target is placed at a certain distance in front of the participant’s eyes, and the power of the negative lens is gradually increased. To maintain the clarity of the vision target, the participant’s eyes were induced to adjust at this time. When the vision target is blurred and its clarity cannot be improved, the participant’s eye can be considered to have reached the maximum accommodation power, which is the AA.

#### NHV

The NHV is measured to determine the horizontal convergence and divergence abilities of participants required to maintain binocular vision (the ability of base out(BO) and base in(BI)) through the use of a prism. A gradual increase in the Prism diopter causes a shift in the horizontal position of the target on the retina, forcing the patient to use a vergence system to compensate for this shift. Prism diopter, marked as ^△^, is the unit used to measure the NHV.

For measuring the NHV, three types of data are required:

1. *Blur point* It means that the participant can no longer use the vergence ability to compensate for the removal of the target from the retina caused by the prism but can still maintain a stable adjustment.
2. *Breaking point* It means that the participant can no longer maintain single binocular vision (SBV) after exhausting all the vergence ability.
3. *Recovery point* It means that the induced removal of the target from the retina caused by the prism is gradually reduced. Thus, the vergence ability can be reused to obtain SBV.

For measuring the NHV, the near target should be placed 40 cm on the near-point rod, with satisfactory lighting. The Risley lens of the comprehensive refractometer is initially placed at the zero (horizontal) position. Subsequently, the prism is increased in front of both eyes at a speed of approximately 1 prism per second. When the participant sees that the target is becoming blurred, it indicates a blur point, and when the visual target changes to two, it refers to the breaking point. When the breaking point appears, the prism was slightly increased in the same direction over the breaking point. Subsequently, the prism is reduced in the opposite direction until the participant reports that the target has become one again, which refers to the recovery point. The sum of the binocular prism power of the blur, rupture, and recovery points was calculated. The entire process was performed using BI and then BO.

In the two aforementioned experiments, we used the NIDEK RT-600 phoropter (comprehensive refractor) to measure the accommodation and vergence ability.

#### IOP

IOP refers to the pressure inside the eyeball. In particular, IOP is the equilibrium pressure exerted by the eye contents on the wall of the eyeball. The IOP of a healthy individual is stable within a certain range, and it functions to maintain the normal shape of the eyeball and ensure that the refractive medium results in the maximum optical performance. The range of normal IOP is 11–21 mmHg.

A tonometer was used to flatten the cornea by using a certain weight to measure IOP according to the flattened corneal area or flattening a certain area of the cornea by using a variable weight. In addition, the tonometer was used to measure IOP according to the required weight. The IOP is directly proportional to the applied external force and inversely proportional to the area of the cornea being flattened.

The Goldmann applanation tonometer (GAT) and noncontact tonometer (NCT) are the two types of applanation tonometers commonly used in clinical practice. The design of both tonometers is based on the Imbert–Fick principle, in which a fixed area is used to measure IOP. The GAT is the gold standard for IOP measurement and is currently the most accurate tonometer with the most favorable design (error range: ± 0.5 mmHg). The NCT does not touch the cornea and can thus prevent iatrogenic cross-infection. Its other advantages include simple operation and easy patient acceptance. Many studies have compared IOP measurements obtained using the GAT with those obtained using the NCT in the healthy general population. No significant difference was observed in the measured values of the GAT and NCT^32^ when the IOP of participants was < 20 mmHg. We used the NIDEK NT-2000 NCT that uses air pressure pulses and considers corneal recovery to obtain measurements.

#### Stereo vision

Stereopsis, the advanced part of human binocular vision function, refers to the depth perception caused by the parallax angle of the binocular. Its unit is the arc angle of second (″). Stereopsis can perceive the three-dimensional ability of various objects in space such as distance, front and back, height, depth, and unevenness.

In this study, we used Randot stereopsis to measure the parallax angle of stereopsis. Randot stereotest uses a circle diagram to examine the stereo vision with a sharpness range of 400″–20″. Polarized glasses must be worn during the examination. The disparity angle should be measured at a distance of 40 cm.

The VRTRID VR headset D601 was used as the Mobile-VR glasses (Fig. 2). The smart phone size ranged from 4.7″–6″. The maximum width and length were 82 and 157 mm, respectively. The field of view and size were 120° and 194 × 129.5 × 109.5 mm, respectively.

### Experimental environment

We simulated a situation wherein a participant wears VR glasses to watch 3D movies. The test location of this study was a bright room, and the indoor temperature was controlled at 25 °C ± 2 °C^33^. The indoor lighting was controlled at 200–400 lx.

### Statistical analysis

We used SPSS version 19.0 for Windows to analyze variations in all parameters. The Shapiro–Wilk test is used to evaluate the distribution of all variables. Normally distributed data is expressed as the mean ± standard deviation, while non-normally distributed data is expressed as the median (interquartile range). The paired t-test was used to analyze variables of normal distribution, and Wilcoxon signed rank test was used to analyze variables of non-normally distributed. Count the correlation between the changes in parameters such as refractive error, amplitude of accommodation, divergence/convergence, stereopsis and IOP before and after using VR. The significance level was set at *P* < 0.05 for all tests.

## Experimental results

### Refractive error examination

**Figure 3:**
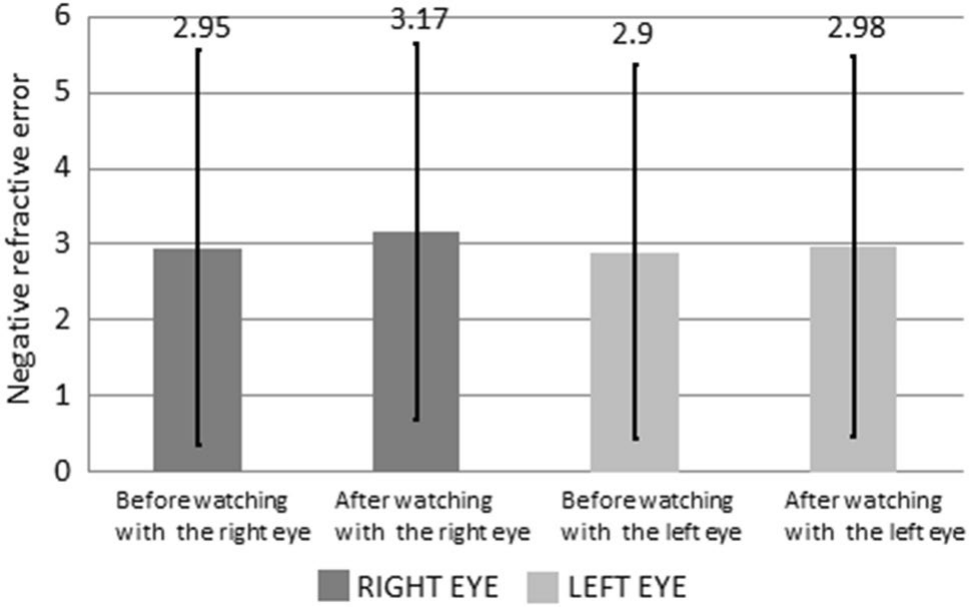
Negative Refractive error before and after the use of VR for watching 3D videos.

Table 1 lists the results of the refractive error and AA examination. Figure 3 presents variations in the refractive error in the left and right eyes of the participants before and after using VR glasses, and the differences were not significant.

**Table 1:** The Refractive error and amplitudes of accommodation changes, in unit D, before and after the use of VR for watching 3D videos.

		Before using VR	After using VR	*P*-Value
Refractive error	Right eye	− 2.95 ± 2.60	− 3.17 ± 2.48	*P* > 0.05
	Left eye	− 2.90 ± 2.47	− 2.98 ± 2.50	*P* > 0.05
AA	Right eye	8.10 ± 1.79	6.27 ± 1.74	*P* < 0.05
	Left eye	7.99 ± 2.48	6.11 ± 1.67	*P* < 0.05

### AA

**Figure 4:**
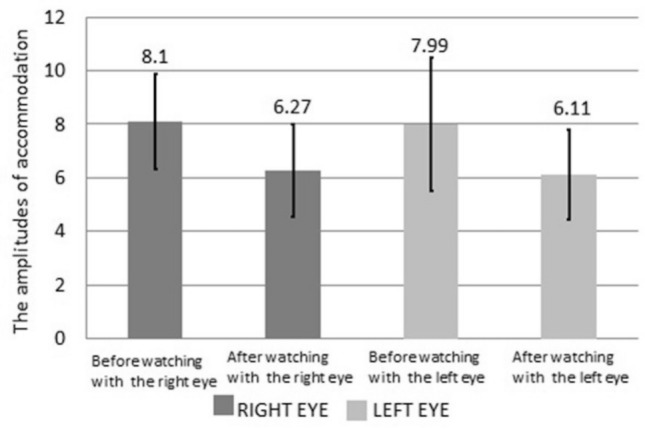
AA before and after the use of VR for watching 3D videos.

Figure 4 presents variation in the AA in the left and right eyes of the participants before and after using VR glasses. The mean AA of the left and right eyes before using VR glasses decreased by 1.83 ± 0.05 D and 1.88 ± 0.81 D, respectively, compared with the values obtained after using VR glasses (*P* < 0.05).

### NHV

**Figure 5:**
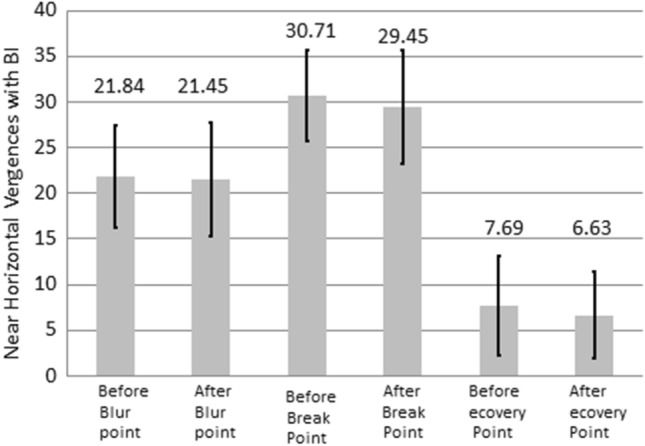
NHV with BI changes before and after using VR for watching 3D film.

**Figure 6:**
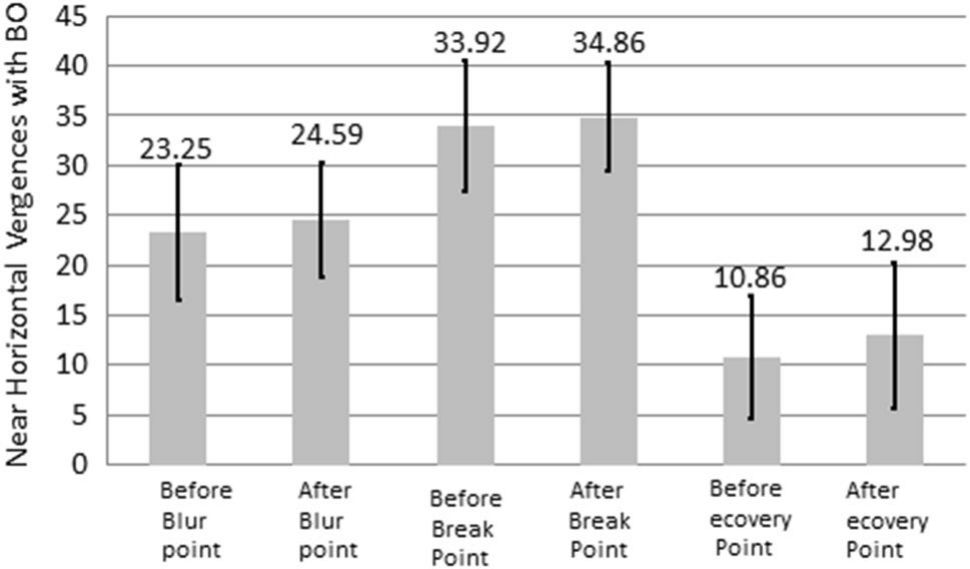
NHV with BO changes before and after using VR for watching 3D film.

Table 2 list the results of the NHV examination. BI and BO represent the divergence and convergence abilities of both eyes, respectively. Figures 5 and 6 show variations in the NHV in the left and right eyes of the participants before and after using VR glasses, and the differences were not significant (*P* > 0.05). However, the center values of the result revealed that the NHV for BI in both eyes decreased after using VR glasses compared with before using VR glasses. However, the NHV for BO in both eyes increased after using VR glasses compared with before using VR glasses.

**Table 2:** The NHV changes with Base in and out, in unit Prism, before and after the use of VR for watching 3D videos.

		Before using VR	After using VR	*P*-Value
Base in	Blur point	21.84 ± 5.57	21.45 ± 6.23	*P* > 0.05
	Breaking point	30.71 ± 5.01	29.45 ± 6.25	*P* > 0.05
	Recovery point	7.69 ± 5.46	6.63 ± 4.76	*P* > 0.05
Base out	Blur point	23.25 ± 6.79	24.59 ± 5.77	*P* > 0.05
	Breaking point	33.92 ± 6.52	34.86 ± 5.48	*P* > 0.05
	Recovery point	10.86 ± .16	12.98 ± 7.24	*P* < 0.05

### IOP

**Figure 7:**
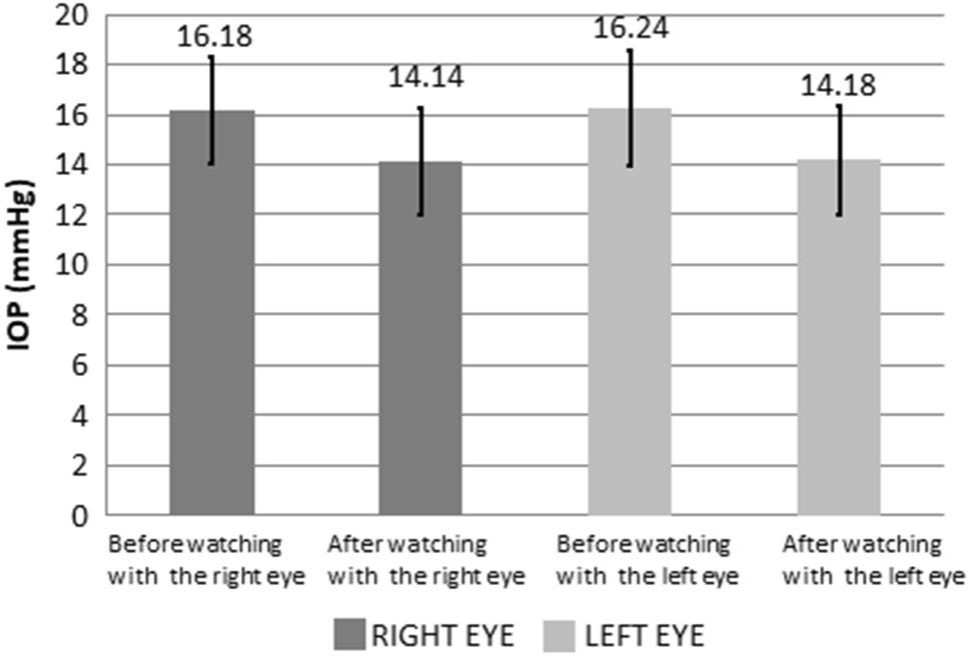
Intraocular pressure changes before and after using VR for watching 3D film.

As presented in Table 3 and Fig. 7, when watching a 3D video, the average IOP values of the right and left eyes were 16.18 ± 2.13 and 16.24 ± 2.29 mmHg, respectively, before using VR glasses and 14.14 ± 2.16 and 14.18 ± 2.17 mmHg, respectively, after using VR glasses. A comparison of IOP values before and after using VR glasses indicated that the right (left) IOP of both eyes decreased by 2.04 ± 0.03 (2.06 ± 0.12) mmHg on average (all *P* < 0.05).

**Table 3:** Intraocular pressure changes, in unit mmHg, before and after the use of VR for watching 3D videos.

	Before using VR	After using VR	*P*
IOP of right eye	16.18 ± 2.13	14.14 ± 2.16	*P* < 0.05
IOP of left eye	16.24 ± 2.29	14.18 ± 2.17	*P* < 0.05

### Stereopsis

**Figure 8:**
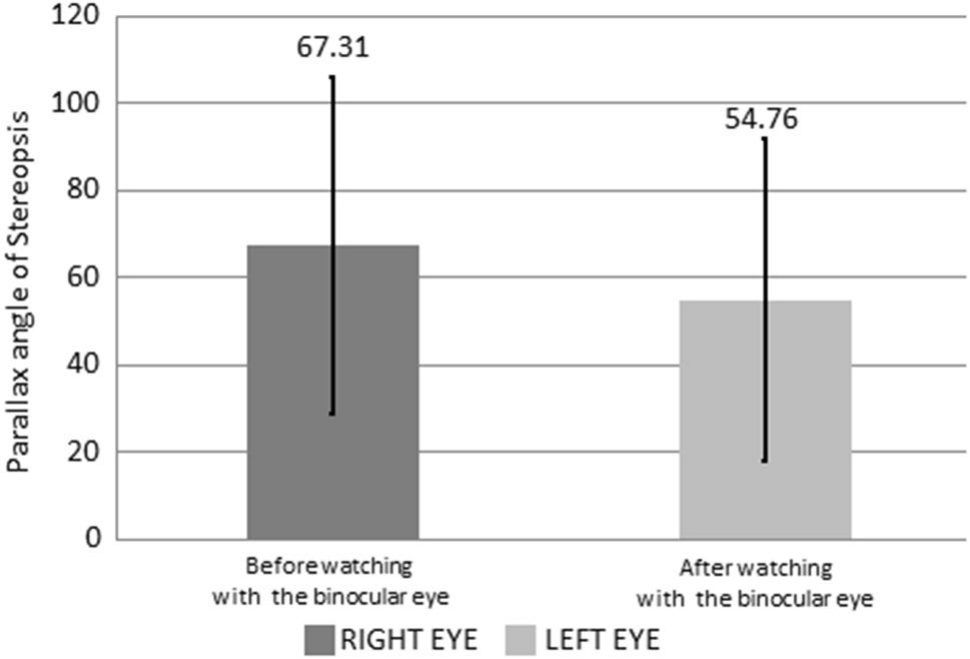
The parallax angle of stereopsis changes before and after using VR for watching 3D film.

The unit of the parallax angle of stereopsis is arc/s. As shown in Table 4 and Fig. 8, when watching a 3D video, the parallax angle values of both eyes were 67.31 ± 38.70 and 54.76 ± 37.05 arc/s before and after using VR glasses, respectively. A comparison of parallax angle values before and after using VR glasses revealed that the stereopsis of both eyes decreased by 12.55 ± 1.65 arc/s on average (all *P* < 0.05).

**Table 4:** The parallax angle of stereopsis changes, in unit arc/sec, before and after the use of VR for watching 3D videos.

	Before using VR	After using VR	*P*-Value
Parallax angle of stereopsis	67.31 ± 38.70	54.76 ± 37.05	*P* < 0.05

## Discussion

In previous research^3–7^, before measuring visual parameters, use immersive VR equipment for at least 30 min, so it is not meaningful to compare with it. The results of the accommodation test revealed that the AA of the participants’ eyes decreased after using VR glasses to watch a 3D video. The mean AA of the participants’ right and left eyes decreased by 1.83 ± 0.05 D and 1.88 ± 0.81 D, respectively, after using VR glasses. On the basis of a previous study^34^, we compared the Hofstetter’s curve of the AA^35^. In teenagers aged 16–18 years, the AA was reduced by approximately 8D after using VR glasses. Therefore, we believe that the participants reached the level of visual fatigue after using VR glasses. This can explain why many people feel uncomfortable after using VR devices.

When we compared IOP values before and after using VR glasses, we observed that the right (left) IOP values of both eyes decreased by 2.04 ± 0.03 (2.06 ± 0.12) mmHg on average. The mechanism underlying the maintenance of IOP mainly involves the smooth circulation of the aqueous humor secreted by the ciliary body inside the eyeball. The aqueous humor can be drained using two methods, which are described as follows.

1. The aqueous humor provides nutrients in the eyeball and is then transported to the lens, iris, and cornea. Metabolites enter the Schlemm canal from the angular trabecular meshwork at the junction of the iris and reach the collection tube and subsequently the anterior ciliary vein.
2. The second method involves the uvea–sclera path: The aqueous humor passes through the ciliary body zone of the anterior chamber angle and enters the gap in the ciliary muscle. It then enters the ciliary body and the suprachoroidal space. Finally, it enters the venous drainage through the blood vessel.

**Figure 9:**
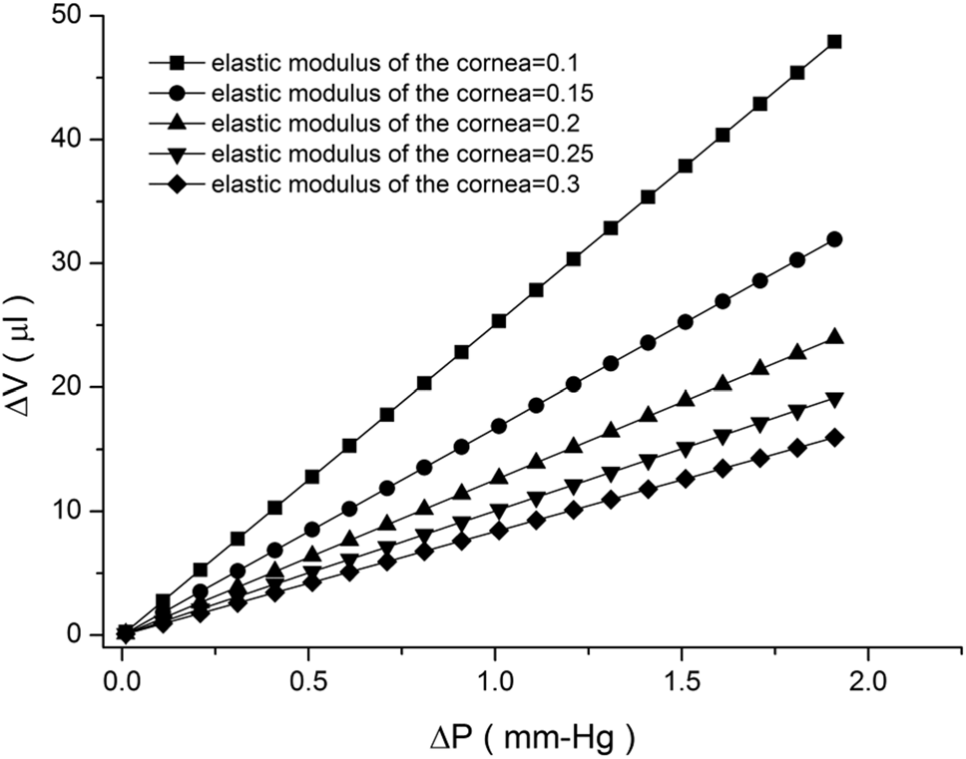
Relationship between IOP change and eyeball volume change under the distinct elastic modulus of the cornea.

Under normal circumstances, a dynamic balance is maintained between the production and discharge of the aqueous humor. However, when excessive aqueous humor is produced or the discharge is blocked, the production of the aqueous humor causes the accumulation of the humor in the eye, thus increasing the IOP, which may suppress the optic nerve and cause visual field defects. Vision is consequently reduced, and symptoms such as the macular degeneration of the retina (which constitutes glaucoma) occur. Therefore, IOP is a crucial parameter for the use of VR. IOP is similar to the pressure in a thin spherical shell in that it can be modeled by mechanically analyzing stress and strain. The relationship between changes in IOP and those in the eyeball volume is discussed later in this paper. Regarding the radial pressure on a thin shell, the stress generated after the change in radial pressure in the thin spherical shell can be calculated as

1 $$\sigma = \frac{\Delta P R}{{2t}}\,\,\,\,\left[ {{36}} \right],$$

where *σ* is the shell volume stress, $\Delta P$ is the change in IOP, R is the sphere radius (referring to that of the cornea and sclera), and *t* is the shell thickness (referring to that of the cornea and sclera). After the eyeball is subjected to stress, the corresponding strain generated can be examined as follows:

2 $$\epsilon_{V} = \frac{\Delta V}{V} = \frac{{3{\upsigma }}}{E}\left( {1 - \nu } \right),$$

where $\epsilon_{V}$ is the volume strain, *ν* is Poisson’s coefficient, and *E* is the elastic modulus (referring to that of the cornea or sclera). Furthermore, *V*, the original volume of eyeball, is denoted as

3 $${\text{V}} = \frac{4\pi }{3}R_{0}^{3} .$$

where *R*_*0*_ is the initial radius without stress. By combining the aforementioned equations, the relationship between changes in IOP and those in the eyeball volume can be investigated (Fig. 9). As shown in Fig. 9, we used R (radius of cornea) = 7.3 mm, *t* (cornea thickness) = 0.55 mm, *ν* = 0.42, and R_0_ = 7.7 mm, respectively. The flow rate of the aqueous humor was relatively stable and not affected by pressure until the pressure reached a considerably high level. The average secretion rate of the aqueous humor was approximately 2.5 μL/min. We compared these characteristics with the results of the experiments presented in Table 3. We propose the following two explanations for the experimental results.

1. The eye volume appeared to change. We expected that the elastic modulus of the cornea would be at least higher than 0.2 MPa (Condition 1).
2. In the 10-min experiment, the eye muscles or other body parts could change IOP (Condition 2).

In this experiment, VR glasses were used to watch 3D movies. The use of VR glasses led to the contraction of the pupils because of the close proximity of the screen to the eye^37^. Moreover, accommodation and convergence must occur for the image to be clearly projected on the retina. During accommodation, the ciliary muscle relaxes, the front and rear diameters of the lens become thicker, the anterior lens capsule moves closer to the cornea, the anterior chamber becomes shallow, and the position of the posterior capsule remains relatively fixed owing to the constant volume of the vitreous; these changes cause an increase in IOP. Simultaneously, due to the strengthening of convergence, the contraction of extraocular muscles directly compress the sclera and increase IOP inside the eyeball. However, the contraction of extraocular muscles can hinder vortex venous return. The contraction of extraocular muscles inhibits the vortex venous return, thus increasing pressure on the suprascleral venous sinus, obstructing the aqueous humor return, and increasing IOP. Thus, IOP decreased when the participants watched a 3D movie. We speculated that when an individual watches a 3D movie, the left and right eyes are used to watch different images, thus resulting in binocular parallax. When the images of the left and right eyes are synthesized by the brain, the distance of the object can be judged according to the size of the parallax^38^; this causes the brain to continually fuse different images provided by both eyes. The VR device only provides movies with a single depth of field, and the movie’s depth of field is fixed. This causes the human eye to always focus on a plane at a fixed distance. In general, when the depth information perceived through focus blur is inconsistent with that perceived through the binocular parallax, severe accommodation–convergence conflict occurs in the brain^39–42^. When this defocusing phenomenon occurs, the optic nerve signal is transmitted to the brain, thus triggering the midbrain Edinger–Westphal nucleus to send a signal to the ciliary nerve. This process causes the ciliary nerve to control the contraction and relaxation of the ciliary muscles. Because the contraction and relaxation of the ciliary muscles cause the dilation and shrinkage of the pupils, respectively, the aqueous humor in the anterior chamber is squeezed into the posterior chamber, thus causing a decrease in IOP. In addition, the contraction of the ciliary muscles causes the contraction of the anterior chamber angle, which causes the aqueous humor to rapidly flow into Schlemm’s tube to reduce IOP. This possibly explains why watching a 3D movie causes a reduction in IOP.

The probability of the occurrence of Condition 2 within 10 min is rather low. Condition 1 is more likely to occur. A slight change in the intraocular volume caused by imbalanced aqueous humor inflow and outflow can change IOP; however, we expected that under such a situation, the elastic modulus of the cornea would be at least higher than 0.2 MPa. Friedenwald^18^ assumed a fixed eye size, changing pressure, and changing volume in his calculations. We used Friedenwald’s ocular rigidity relationship in our study:

4 $$\ln P_{2} - \ln P_{1} = K \Delta V.$$

**Figure 10:**
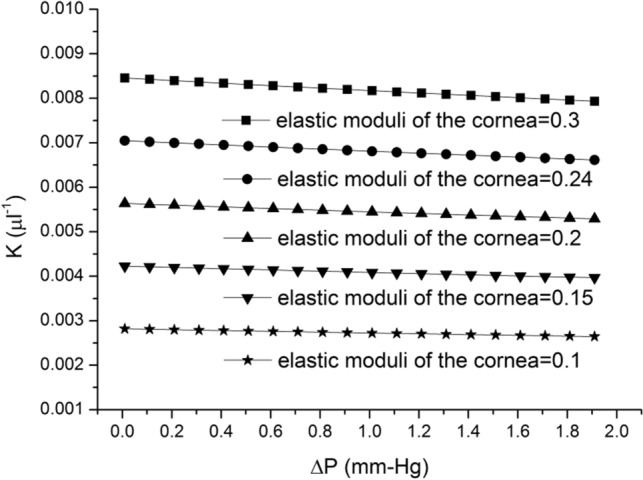
Relationship between IOP changes and K values under different elastic moduli of cornea.

Equations (1)–(4) were combined to investigate the relationship between IOP changes and *K* values under different elastic modulus values of the cornea (Fig. 10). When the elastic modulus of the cornea was higher than 0.2 MPa, the *K* value was approximately 0.0055–0.0090, which is close to the results *K* ~ 0.008–0.011 obtained by Woo^20^. Compared with the *K* ~ 0.0024 reported by Friedenwald^18^ and Ridley^19^, the value obtained in the present study was almost two times larger. But, if we use 0.1 MPa for our elastic modulus of the cornea, it will be similar to their results. This result revealed that together with volume changes with small IOP variations, the pressure increased linearly. This finding is equivalent to the value obtained when the human eyes were placed under pressure in the range used in a previous study^20^. Visual fatigue and discomfort caused after using VR glasses to watch 3D movies^41^ remain key issues. Therefore, IOP measurements obtained after using VR glasses can provide a basis for preventing fatigue and discomfort caused by the use of VR glasses among teenagers. In addition, our findings provide insights into physiological parameters that may be used to improve the treatment compliance of glaucoma in early stage or under good disease control^44^.

In this study, we observed that the stereopsis of both eyes of the participants increased after using VR glasses to watch a 3D video. Before and after viewing 3D video in VR, the parallax angle of both eyes decreased by 12.55 ± 1.65 arc/s. Because stereo vision can distinguish the spatial position of an object, including the relative position of front and rear, distance, and height, it can also distinguish the minimum binocular parallax angle of other people’s eyes. Therefore, the smaller the stereopsis values are, the smaller is the parallax angle and the better is the stereo vision. As shown in Table 2, the central value of BO in the NHV test was observed to be higher although the result of the paired-sample t test showed no significant difference. This may be attributable to the fact that the time of the experiment was not long enough, thus resulting in nonsignificant differences. This can be seen in the report of Alvin J. Munsamy^4^, after using VR for 25 min, the vergence ability of the eyes has indeed increased. Because the distance between the eyes and VR glasses was only 15 cm, we believe that the convergence ability of the eyes would increase after using VR glasses. In the 40-cm stereoscopic test, the eyes can more easily converge to distinguish subtle changes in the picture, thus resulting in the reduction of the resolution angle of the eyes and an increase in the stereoscopic ability. This result is different from Mon-Williams^13^, in their report that there is no significant change in the average stereo vision, which may be related to only two subjects. Another problem is that a higher convergence ability would be required when viewing nearby objects in VR than when viewing objects in the real world. Therefore, individuals with abnormal binocular vision, such as those who may already have convergence excess, may still have symptoms.

Cybersickness symptoms are unavoidable problems encountered while using mobile devices with VR. In this study, the participants were allowed to use mobile VR only for 10 min, so they did not have a considerable sense of immersion and spatial dislocation. Therefore, we do not need to consider the cognitive and neuropsychological problems that arise from using VR. However, like Mon-Williams^13^, approximately 10% of the participants still experienced cybersickness symptoms such as nausea, dizziness, disorientation (motion sickness), fatigue, and instability, likely due to binocular parallax^45^. Although binocular parallax is the main condition for stereoscopic vision, cybersickness symptoms are enhanced under stereoscopic vision conditions^46–48^. This is because a difference is noted between the two eyes while watching a VR movie. At this time, for the purpose of fusion, the eyes will be in a state of rapid and uncoordinated accommodation and convergence or divergence; this can cause cybersickness symptoms such as nausea, dizziness, and eye fatigue^49–51^. Participants can have a considerable degree of immersion as soon as possible, which may be a good option (although it will induce cybersickness symptoms, the period of VR use would be longer^52^). Because users have a sense of spatial dislocation and depth after a considerable degree of immersion in VR, this can prevent the eyes from experiencing increased proximal accommodation and convergence, resulting in the nonrequirement of a reduction in the AA and considerable convergence would not be required for generating stereoscopic vision. Hence, mobile devices with VR must have higher quality and comfort in the following ways. First, the lens in the device should be appropriately designed to reduce the eye position movement caused by the accommodation and convergence or divergence of the eyes. This can be found in Ref.^3^, whether in the exophoric and esophoric direction, the heterotropia has changed. The eye position movement can cause the lens to produce a prism effect and a change in the equivalent diopter. Second, the accommodation and convergence or divergence of the eyes are coupled and linked. Therefore, for the coordination of the accommodation and convergence or divergence of the eyes while using VR, movies with a higher quality and flicker rate are necessary. In addition, the scene design of the film should be considered. Third, appropriate visual training (i.e., some training courses using VR) should be developed.

## Conclusion

According to Ref.^3^, using a VR headset for 40 min does not seem to affect the binocular vision. After using the VR headset for 10 min, by measuring IOP and some visual parameters of the eyes, the diopter and divergence abilities of the eyes at a close range appeared to have no effect. However, changes could be observed in IOP, AA, and stereopsis. This shows that at the beginning of using VR, it did have an impact on the accommodation and convergence of the eyes, and also changed the stereoscopic vision. In summary, 3D VR is a very promising new tool. It may be used to control glaucoma and help diseases such as insufficient binocular convergence and poor stereo vision, it can be used as a tool for vision training. The report in Ref.^53^ pointed out that adults have a significant increase in the accommodation range after a period of treatment using VR, which shows that the accommodation ability of the ciliary muscle and lens is strengthened, and it is not easy to cause visual fatigue. Additional studies are required to introduce this technology into clinical practice in the future^54,55^. For some VR-user who have symptoms of early-onset nausea, dizziness, and eye fatigue, it may be a good way to develop a considerable degree of immersion as soon as possible. Our data can help designers design more comfortable VR glasses.

### Ethical approval

All the authors’institutions have not established the institutional review board (IRB).According to Chapter 2 Article 5 of the Taiwan Human Body Research Act, prior to conductinga study, the principal investigator should submit the research protocol for review and approval by the IRB. The review in the preceding paragraph shall be conducted by the research entity’s IRB. Where an entity does not have an established IRB, the review may be conducted by the IRB of other entity. In this study, all procedures performed were in accordance with the ethical standards of the Human Research Ethics Committee of National Cheng Kung University (approval No. NCKU HREC-E-107–315-2) and with the 1964 Helsinki declaration and its later amendments or comparable ethical standards.

### Informed consent

Informed consent was obtained from all patients.
